# PERCEIVED FATIGABILITY AND MORTALITY IN MID-TO-LATE LIFE IN THE BALTIMORE LONGITUDINAL STUDY OF AGING

**DOI:** 10.1093/geroni/igac059.1459

**Published:** 2022-12-20

**Authors:** Amal Wanigatunga, Xiaomeng Chen, Francesca Marino, Eleanor Simonsick, Luigi Ferrucci, Jennifer Schrack

**Affiliations:** Johns Hopkins Bloomberg School of Public Health, Baltimore, Maryland, United States; Johns Hopkins School of Medicine, Baltimore, Maryland, United States; Johns Hopkins Bloomberg School of Public Health, Washington, District of Columbia, United States; National Institute on Aging, Baltimore, Maryland, United States; National Institute on Aging, Baltimore, Maryland, United States; Johns Hopkins Bloomberg School of Public Health, Baltimore, Maryland, United States

## Abstract

This study hypothesized that perceived fatigability, fatigue standardized to walking, would better discriminate mortality risk than general fatigue symptoms. We leveraged perceived fatigability, self-reported tiredness, self-reported low energy and mortality data from 1,112 participants aged ≥50 years. Hazard ratios (HRs) of all-cause mortality (198 [17.8%] events) by either z-scored fatigability, tiredness, and energy level were estimated using Cox proportional hazards models. Model discrimination was compared using Harrell’s C-statistics (C). After full covariate adjustment, 1 SD higher perceived fatigability, tiredness and low energy level were associated with higher mortality risk (HR=1.20, 95% confidence interval [CI]:1.05-1.37; HR=1.28, 95% CI:1.11-1.48; HR=1.26, 95% CI:1.10-1.45, respectively). The model for perceived fatigability (C=0.8039) had comparable discrimination to tiredness (C=0.8085,p=0.26) and low energy level (C=0.8067,p=0.41). While perceived fatigability was associated with higher mortality risk, it did not exhibit superior ability to discriminate mortality risk than self-reported fatigue symptoms among persons in mid-to-late life.

